# Long Noncoding RNA LINC01554 as a Novel Biomarker for Diagnosis and Prognosis Prediction of Epithelial Ovarian Cancer

**DOI:** 10.1155/2021/1244612

**Published:** 2021-08-10

**Authors:** Ting Luo, Yan Jiang, Jing Yang

**Affiliations:** Department of Obstetrics and Gynecology, The Renmin Hospital of Wuhan University, Wuhan, Hubei 430060, China

## Abstract

**Objective:**

This study was aimed at exploring the diagnostic and prognostic value of long noncoding RNA LINC01554 (LINC01554) in epithelial ovarian cancer (EOC) patients. *Patients and Methods*. The expressions of LINC01554 in 161 EOC patients were analyzed using RT-PCR. The area under the ROC curve (AUC) was used to estimate the effectiveness of LINC01554 for prediction. The chi-square test was performed to explore the association between LINC01554 expressions and clinical characteristics in EOC patients. Kaplan-Meier assays were conducted for the examination of the influence of LINC01554 expression on the overall survival of EOC patients. Multivariate analyses were carried out to further determine prognostic values of LINC01554 expression in EOC patients.

**Results:**

LINC01554 expressions were strongly downregulated in EOC specimens compared with matched nontumor specimens (*p* < 0.01). Importantly, LINC01554 provided a high diagnostic performance for the detection of EOC specimens (AUC = 0.7827; *p* < 0.001). Low expression of LINC01554 was distinctly associated with the FIGO stage (*p* = 0.034) and distant metastasis (*p* = 0.007). The assays of survival data (five years) revealed that the 5-year overall survival of the low LINC01554 expression group was distinctly shorter than that of the high LINC01554 expression group (*p* = 0.0017). Finally, in the multivariate Cox model, LINC01554 expression (RR = 2.863, 95% CI: 1.185-4.421, *p* = 0.014) was demonstrated to be an independent prognostic factor for overall survival of EOC patients.

**Conclusions:**

Our findings suggested that LINC01554 is an important EOC-related lncRNA, providing a potential diagnostic, prognostic biomarker and therapeutic target for EOC patients.

## 1. Introduction

Epithelial ovarian cancer (EOC) accounts for >85% of all ovarian tumor cases, which is still a major cause of morbidity and mortality and the most lethal gynecologic neoplasm all over the world [1, 2]. Each year, >235,000 new cases are estimated to be diagnosed, resulting in at least 110,000 deaths in the world [3]. Although the advances in surgery and chemotherapeutic agents have significantly improved the quality of life of EOC patients, the 5-year survival rate in EOC with advanced stages remains only 30% due to the high rate of recurrence and metastasis [4, 5]. Thus, the molecular mechanisms underlying EOC progression need to be elucidated for the identification of novel EOC-specific biomarkers for early diagnosis of this tumor.

Long noncoding RNAs (lncRNAs), frequently dismissed as nonfunctional transcriptional “noise,” are RNAs with >200 nucleotides in length with limited protein-coding capacity [6]. Growing studies indicate that lncRNAs contribute to different cellular processes via regulating the expression of various genes in transcriptional, posttranscriptional, or posttranslational levels [7]. Interestingly, a large number of tumor-associated lncRNAs have been characterized, and their potential roles and underlying molecular mechanisms involved in the progression and tumor metastasis have been confirmed in both *in vitro* and *in vivo* assays [6]. More importantly, several lncRNAs have been demonstrated to be detectable in serum and serve as novel sensitive prognostic and diagnostic biomarkers for various tumors, such as lncRNA DLEU1 for bladder cancer, lncRNA BRE-AS1 for prostate carcinoma, and lncRNA TC0101441 for EOC [8–10].

lncRNA LINC01554 (LINC01554), a recently identified tumor-related lncRNA, has been reported to be dysregulated in several types of neoplasms, such as esophageal cancer, cervical cancer, and hepatocellular carcinoma [11–13]. However, the present evidence of abnormal LINC01554 expression in above tumors was limited. For the first time, Zheng et al. [13] performed functional assays, providing robust evidence that downregulation of LINC01554 weakened the proliferation and metastasis of hepatocellular tumor cells via Akt/mTOR signaling. However, the function of LINC01554 in other tumors remained largely unclear. Here, we firstly reported the possible clinical significance of LINC01554 in EOC patients.

## 2. Patients and Methods

### 2.1. Patients and Tissue Samples

161 pairs of fresh EOC specimens and their adjacent normal specimens were obtained from 161 EOC patients (age: 23-68) from the Renmin Hospital of Wuhan University with approval from the Ethics Committee of our hospital. The diagnosis of EOC was confirmed pathologically, and all samples in the histological characterization and clinicopathological staging were determined. The clinical information of all cases was recorded by using an electronic file with a long-term follow-up more than five years. Prior patients' written informed consent was obtained, which allowed us to use their clinical materials for research purposes.

### 2.2. RNA Extraction and qRT-PCR Analyses

Using TRIzol based on the manufacturer's protocol, total RNA was extracted from all samples. A total of 600 ng RNA was converted into cDNA using the PrimeScript™ RT reagent kit (Takara, Suzhou, Jiangsu, China). RT-PCR was carried out using the SYBR Green PCR Kit (Takara, Suzhou, Jiangsu, China) on the ABI 7500 Fast Real-Time PCR System (Biosystems, USA). Relative expression values were calculated by the 2^−ΔΔCt^ methods. GAPDH was used as an internal reference. The primers used in this study were shown below: for LINC01554, CATGGAGTATCTAAGCAGCC (forward) and CAGTTGAYAGCGGAGCCTTG (reverse) and for GAPDH, CAATGACCCCTTCATTGACC (forward) and GACAAGCTTCCCGTTCTCAG (reverse).

### 2.3. Statistical Analysis

All data were statistically analyzed using SPSS (19.0 version; IBM, Chicago, IL, USA). Differences between groups were analyzed using Student's *t*-test or one-way ANOVA based on the types of groups. ROC assays were carried out to explore the diagnostic value of LINC01554 for EOC patients. By the use of the Kaplan-Meier method with the log-rank test, our group calculated the overall survival rates of all patients. Multivariate assays were carried out to test the prognostic value of LINC01554 in EOC patients. *p* < 0.05 was considered statistically significant.

## 3. Results

### 3.1. Low Expression of LINC01554 in EOC Patients

**Figure 1:**
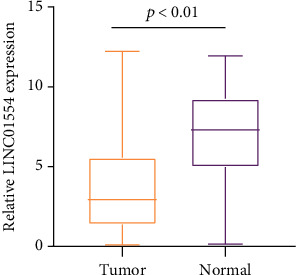
RT-PCR for the expression of lncRNA LINC01554 in specimens compared to matched nontumor tissues.

In recent years, more and more lncRNAs were identified to be dysregulated in EOC. To explore whether LINC01554 was a functional lncRNA in EOC progression, we performed RT-PCR to examine its expression in 161 EOC specimens. As presented in Figure 1, LINC01554 levels in EOC specimens were distinctly lower than those in noncancerous tissues (*p* < 0.01).

### 3.2. The Diagnostic Value of Dysregulated LINC01554 Expression in EOC Patients

**Figure 2:**
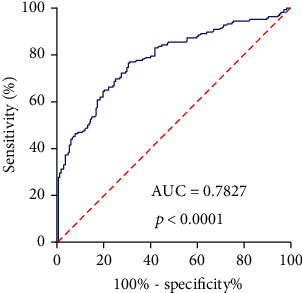
ROC curve of lncRNA LINC01554 expression to distinguish EOC specimens from matched nontumor tissues. The area under the curve (AUC) of lncRNA LINC01554 was 0.7827.

To examine whether the tissue LINC01554 had diagnostic potential, we performed ROC assays. As shown in Figure 2, LINC01554 had an AUC value of 0.7827 (95% CI 0.7333 to 0.8322) for EOC. In addition, the sensitivity and specificity of LINC01554 for distinguishing EOC specimens from nontumor tissues was 73.32% and 89.67%, respectively. Overall, our findings revealed that LINC01554 may be used as a biomarker for the early screening of EOC patients.

### 3.3. Association between LINC01554 Expression and Clinical Features of EOC Patients

The median value of relative LINC01554 expression was 6.43 according to the test of qRT-PCR in 161 samples of EOC, which were classified into two groups (high group: *n* = 80, low group: *n* = 81). Then, the chi-square test was performed, which indicated that low LINC01554 expressions were associated with the FIGO stage (*p* = 0.034) and distant metastasis (*p* = 0.007, Table 1). However, LINC01554 expressions in EOC were not correlated with other parameters (all *p* > 0.05).

### 3.4. Low Expression of LINC01554 Correlates with Poor Prognoses

**Figure 3:**
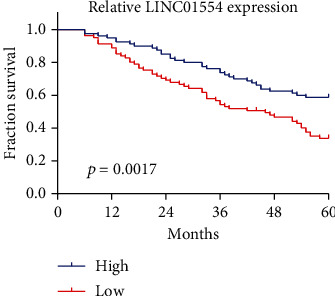
Kaplan-Meier survival curves for EOC patients according to lncRNA LINC01554 expression.

In the above findings, we confirmed that patients with low LINC01554 expression displayed a positive metastasis, suggesting that LINC01554 may influence the prognosis of EOC patients. We collected information on 5-year survivors from 161 EOC patients and performed Kaplan-Meier's assays. As presented in Figure 3, we observed that the 5-year overall survival of the low LINC01554 expression group was distinctly shorter than that of the high LINC01554 expression group (*p* < 0.0017). Moreover, in a multivariate Cox model, LINC01554 expression was confirmed to be an independent poor prognostic factor for overall survival of EOC patients (*p* = 0.014, Table 2).

## 4. Discussion

EOC has a high prevalence in China and ranks as one of the most relentless types of tumors disturbing the female reproductive tract [14]. Clinical reports based on several groups have suggested that the majority of EOC patients have achieved a favorable outcome due to the early diagnosis which possesses important abilities contributing to the systematic optimization of therapeutic schedules [15, 16]. Despite the fact that several clinicopathological indexes have been applied to screen EOC patients, these factors have limited specificity and sensitivity, which require further improvements [17, 18]. Growing studies have indicated that some lncRNAs were distinctly dysregulated in various tumors, and a large number of functional assays have confirmed lncRNAs as potential participants in cancer development via acting as tumor suppressors for oncogenes, which highlighted their potential use as novel diagnostic and prognostic biomarkers for tumor patients [19, 20].

LINC01554 was a recently identified lncRNA whose upregulation was firstly confirmed in hepatocellular carcinoma according to a previously published microarray analysis [21]. Then, a previous study by Zheng et al. [13] further provided similar evidence that LINC01554 was lowly expressed in hepatocellular carcinoma by performing RT-PCR in 167 primary hepatocellular carcinoma samples. Importantly, they showed that knockdown of LINC01554 suppressed the proliferation and metastasis via suppressing the Akt/mTOR signaling pathway, suggesting LINC01554 as an oncogenic lncRNA in hepatocellular carcinoma. On the other hand, a clinical study by Ding et al. [22] confirmed that low LINC01554 expression predicted a poor prognosis of hepatocellular cancer. These findings suggested LINC01554 as a tumor promotor in hepatocellular cancer progression. However, the function of LINC01554 in other tumors has not been investigated.

In this study, for the first time, we confirmed that LINC01554 was distinctly downregulated in EOC, which was in line with its expressing trend in hepatocellular carcinoma. Then, we analyzed its clinical significance in our cohort, demonstrating that lower LINC01554 expressions in EOC specimens were associated with the FIGO stage and distant metastasis. Due to the prognostic value of the positive metastasis in EOC patients, we wondered whether LINC01554 could modulate the clinical outcomes of EOC patients. Using Kaplan-Meier methods, we found that EOC patients with high levels of LINC01554 suggested a short survival time in comparison with those with low levels of LINC01554. More importantly, the results of multivariate analyses confirmed LINC01554 as an independent poor prognostic factor for overall survival of EOC patients. Although our findings provided clinical evidence on the clinical values of LINC01554 in EOC patients, the sample size is relatively small, and large clinical trials are needed to be conducted. In addition, based on our results, we suggested LINC01554 as a tumor-suppressive lncRNA in EOC. Thus, in vitro and in vivo assays with gain-of and lost-of-function experiments were needed to explore the specific function of LINC01554 in EOC cells.

## 5. Conclusions

We revealed that LINC01554 may be a novel diagnostic and prognostic biomarker for EOC patients.

## Figures and Tables

**Table 1:** Correlation between LINC01554 expression and clinicopathological features in EOC patients.

Parameters	Group	Total	LINC01554 expression		*p* value
Age (years)	<55	79	35	44	0.180
	≥55	82	45	37	
Histological subtypes	Serous	88	48	40	0.721
	Endometrioid	23	9	14	
	Mucinous	19	11	8	
	Clear cell	17	7	10	
	Others	14	5	9	
Tumor size (cm)	≤5	110	59	51	0.141
	>5	51	21	30	
FIGO stage	I+II	108	60	48	0.034
	III+IV	53	20	33	
Grade	G1	108	57	51	0.263
	G2+G3	53	23	30	
Distant metastasis	Yes	39	12	27	0.007
	No	122	68	54	

**Table 2:** Univariate and multivariate analyses of prognostic parameters in EOC patients by Cox regression analysis.

Variable	Univariate analysis			Multivariate analysis		
	RR	95% CI	*p* value	RR	95% CI	*p* value
Age (years)	1.328	0.667-1.832	0.323	—	—	—
≥55 vs. <55						
Histologic grade	1.441	0.722-1.933	0.286	—	—	—
Serous vs. endometrioid+mucinous+clear cell+others						
Tumor size (cm)	1.399	0.873-2.218	0.185	—	—	—
≤5 vs. >5						
Tumor grade	1.311	0.765-1.993	0.121	—	—	—
G1 vs. G2+G3						
Distant metastasis	3.219	1.378-5.281	0.007	2.985	1.173-4.772	0.013
Yes vs. no						
FIGO stage	2.985	1.487-4.775	0.011	2.765	1.285-4.554	0.016
I+II vs. III+IV						
LINC01554 expression	3.019	1.375-4.776	0.009	2.863	1.185-4.421	0.014
High vs. low						

## Data Availability

The data used to support the findings of the present study are available from the corresponding author upon reasonable request.
